# Catalyzed hydrothermal carbonization of sewage sludge: structural modification of hydrochar and its derived selective pyrolytic product distribution

**DOI:** 10.1039/d5ra06935a

**Published:** 2025-11-11

**Authors:** Muhammad Rizwan, Xiaolong Zhou, Asma Leghari, Muhammad Sarfraz Akram, Hassan Zeb, Muhammad Frayad Ali, Muhammad Kashif Javed, Mingzhi Wang, Muhammad Asif Nawaz

**Affiliations:** a International Joint Research Center for Green Energy and Chemical Industry, East China University of Science & Technology Shanghai 200237 China xiaolong@ecust.edu.cn; b Institute of Clean Coal Technology, East China University of Science and Technology Shanghai 200237 China; c Institute of Energy & Environmental Engineering, University of the Punjab Lahore 54590 Pakistan; d State Key Laboratory of Chemical Engineering, School of Chemical Engineering & Technology, East China University of Science & Technology Shanghai 200237 China; e Department of Inorganic Chemistry and Material Sciences Institute of Seville, University of Seville-CSIC Seville 41092 Spain mnawaz@us.es

## Abstract

The pressing demand for sustainable alternatives to fossil fuels coupled with environmental risks associated with inappropriate sewage sludge (SS) disposal calls for innovative valorization strategies that transform waste into value-added products. This study introduces a novel approach by directly incorporating zeolite catalysts (HZSM-5 and USY) into the hydrothermal carbonization (HTC) of SS, followed by pyrolysis (Py) of the derived hydrochar (HC). Insights into derived HC are explored through comprehensive characterization, such as morphology, crystallinity, functionality, and thermal analysis. HZSM-5 significantly reduced the activation energy of HC from 27 to 5.5 kJ mol^−1^, while increasing the structural disorder (ID/IG0.73). The selective production of CO and H_2_ was achieved through temperature-dependent pyrolysis between 500 and 900 °C. HZSM-5 facilitated an increase in CO production to 54.18%, whereas USY boosted CO yield up to 35.6%. The optimal product distribution was achieved by strategically incorporating zeolite catalysts, allowing for precise control of N and O functionality and promoting selective syngas and chemical precursors yield. This innovative catalyst-mediated HTC-Py cascade offers unique control over pyrolytic products by introducing an efficient pathway for transforming problematic SS into green energy carriers, thus bridging the gap between environmental sustainability and feasible industrial utilization.

## Abstract 2

Catalyzed (zeolites) HTC conversion of SS was investigated. Temperature-dependent pyrolysis of catalyzed HC facilitated selective syngas yield. Integrated HTC-Py promoted hazardous waste into valuable chemical precursors.

## Graphical abstract

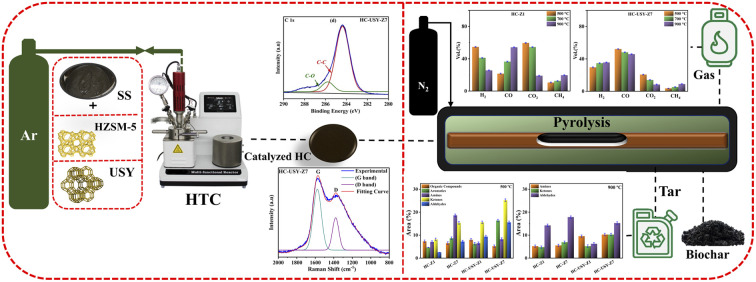

## 1. Introduction

The depletion of fossil fuel reserves, intensified climate changes due to global warming,^1^ and escalated energy demands in urban areas make it imperative to investigate eco-friendly renewable alternatives.^2^ The primary source of diverse hydrocarbon spectra, which are essential precursors in various industrial applications, is non-sustainable fossil feedstocks.^3^ This critical juncture necessitates novel valorization strategies that transform abundant renewable biomass resources into valuable energy carriers and chemical precursors, including light aromatics, effectively connecting a sustainable environment with future-oriented industrial feedstock requirements.^4^ In the diversified biomass landscape, SS is a relatively abundant resource, growing with wastewater treatment facilities caused by the global population. The annual SS production in China was reported to have reached 60 million tonnes,^5,6^ with its handling cost accounting for 30% to 50% of the overall expenses related to wastewater treatment plants.^7^ The improper disposal of this prevailing waste stream poses risks to ecological integrity and public health due to its complex array of contaminants, including heavy metals (HMs), microorganisms, microplastics, and organic pollutants, while holding unexplored renewable resource potential. Conventionally handling approaches, such as landfilling and incineration,^8^ are becoming unsustainable due to environmental concerns and economic issues.^9,10^ SS, abundant in organic matter, catalytic alkali, and alkaline earth metals (AAEMs), serves as a valuable feedstock for bioenergy conversion through biochemical processes, notably anaerobic digestion, achieving 30–60% conversion to biogas within 10–30 days while on the other hand, thermochemical conversion (TCC) processes are more efficient in recovering energy, reducing volume, and eliminating pathogens.^11^ Among the various TCC techniques, gasification, pyrolysis, and torrefaction are some of the notable processes that offer efficient conversion of SS into bioenergy;^9^ however, the paradigm is shifting towards exploring its pretreatment techniques^12^ prior to pyrolysis and gasification because of its inherent significant moisture content and high ash content^13^ which inhibit its processing for effective clean energy generation.

HTC has emerged as a remarkable pretreatment process for efficiently converting high moisture biomass feedstock into valuable HC at moderate temperatures^14^ through dehydration, decarboxylation, and condensation reactions under subcritical water conditions (180–250 °C). Cui *et al.* (2025) also reported that subcritical water converts bound water into removable water, enhancing sludge dewatering *via* structural modification^15^ by eliminating the need for energy-intensive drying of wet biomass and prevents gas-phase emissions by dissolving oxides in the liquid phase.^16^ The *in situ* autogenous pressure promotes hydrolysis of biopolymers such as cellulose, proteins, and lipids, facilitating the formation of stable aromatic and oxygen-depleted carbon structures. Compared with pyrolysis alone, catalytic hydrolysis offers mechanistic advantages including high carbon yield, the ability to treat high-moisture feedstocks without prior drying, and a milder reaction environment that enhances control over surface oxygen functionalities and energy densification.^17,18^ This advanced TCC process turns biomass into carbon-enriched HC with optimized fuel properties and lower oxygen content, providing a versatile platform for valorization. HC is a key component in advanced waste-to-energy cascades, reducing waste volume, eliminating pathogens, and increasing energy density. This clearly demonstrates the promising role of HTC in sustainable economy frameworks that turn problematic wastes into productive energy materials.

Recently, biochar-based composites^19,20^ have gained attention as efficient heterogeneous catalysts^21^ owing to their high surface area, tunable surface functionalities, and strong metal-binding capacity. Studies have shown that biocatalysts^22,23^ and metal-doped systems can significantly enhance catalytic activity in biomass conversion and hydrothermal processes by facilitating deoxygenation and aromatic condensation reactions.^24^ Zeolites function as remarkable catalysts in TCC processes due to their unique characteristics: thermal stability, shape selectivity, ion exchange capabilities, and high specific surface area, resulting in enhanced bio-oil yields and selective biomass pyrolysis.^25^ In recent years, the use of heterogeneous catalysts in HTC has gained significant interest for improving reaction efficiency, HC yield, and physicochemical properties. Modified zeolites (*e.g.*, ZSM-5, Ce/H-ZSM-5) and metal-doped carbon materials (*e.g.*, Fe, Cu, Ni) accelerate dehydration, decarboxylation, and aromatization, enhancing carbon enrichment and oxygen-deficient aromatic structures. Zeolitic frameworks reshape carbon distribution, promote aromatic condensation, and increase energy density, while Cu-/Fe-doped HCs^26^ improve redox behavior, catalytic activity, and structural ordering under subcritical water conditions. For instance, Peng *et al.* (2018) demonstrated that ZSM-5 facilitated aromatic carbon formation and reduced oxygenated intermediates during the HTC of SS.^27^ Similarly, Rasaq *et al.* (2024)^28^ highlighted that zeolite- and metal-assisted HTC systems substantially improve carbonization kinetics and HC stability through enhanced catalytic deoxygenation. Furthermore, Djandja *et al.*, 2023 (ref. 29) comprehensively reviewed catalytic HTC of organic wastes, emphasizing the roles of Fe, Cu, and Ni-based catalysts in improving reaction selectivity and promoting oxygen removal.

Conventional catalytic carbonization processes that utilize zeolites generally function at temperatures exceeding 300 °C and involve intricate deoxygenation pathways, including reactions of oxygen-containing compounds and deamination and deamidation processes.^30–32^ Additionally, synthetically produced zeolites are recognized as paramount catalysts in chemical conversion processes, such as catalytic cracking and the conversion of biomass into chemicals.^33^ It has been demonstrated that the incorporation of various zeolite catalysts improves the efficiency of char and facilitates biomass decomposition. Pyrolysis' ability to convert organic matter into bio-crude, carbonaceous residue, and syngas makes it a key technology for renewable resource utilization and circular bioeconomy initiatives.^34^ Recent studies highlight that HTC products are the ideal feedstock for thermal degradation.^35^ Hydrothermal treatment improves porosity, mineral content, and thermal stability compared to directly pyrolyzed materials.^36^ These improved physicochemical properties greatly expand the multiple applications of hydrothermally processed products. Researchers are focusing on integrated HTC-Py cascades for biomass valorization, which showcases a two-stage strategy by optimizing energy efficiency and resource recovery while converting biomass into high-value products using complementary TCC mechanisms.^37^ Liu *et al.* (2020) investigated the sequential pretreatment of SS combined with pyrolysis, which yielded high-quality bio-oil with improved thermal stability.^38^ Zhang *et al.* (2022) found that HZSM-5 improved the catalytic pyrolytic product distribution of hydrothermally treated kitchen waste HC.^36^ Liu *et al.* (2023) proposed a combined HTC-Py and reforming process for SS conversion to 61.16 vol% H_2_-rich gas.^11^ While pyrolysis catalysts are thoroughly investigated, the catalytic potential of zeolites like HZSM-5 and USY during HTC is seldom addressed. Strategic zeolite addition during HTC could change HC characteristics, reaction kinetics, and downstream pyrolysis performance. However, HTC's research on SS has mainly focused on non-catalytic processes. Zeolites influence product structure and reactivity, but their effect on complex, heterogeneous feedstocks like SS in catalyzed HTC-Py systems is unknown. SS-derived HC and zeolite catalysts during the HTC stage may affect char reactivity, thermal behavior, and syngas/bio-oil yields in pyrolysis; however, this aspect has not been investigated in waste valorization research.

Unlike conventional approaches that apply catalysts during pyrolysis, this work investigates a critical research frontier by comprehensively examining the catalyzed HC and its derivatives through pyrolysis, revealing pathways to optimize their environmental value in advanced applications. This study also extends conventional non-catalytic HTC research by examining the interaction between SS and zeolite catalysts to produce catalyzed HC. The comparative assessment of medium- and large-pore zeolites further provides new insights into pore structure–function relationships under hydrothermal conditions. The primary objective is to (I) provide a comprehensive understanding of physicochemical properties of HZSM-5/USY catalyzed HC, (II) elucidate the thermal behavior of catalyzed HC, thus advancing our understanding of catalytic HTC processes, and (III) to evaluate integrated HTC-Py products distribution (bio-oil and bio-gas) at varied temperature between 500–900 °C.

## 2. Materials and method

### 2.1. Materials

Secondary dewatered SS (80% moisture) was procured from Jiangsu Huineng Environmental Technology Co., Ltd. The SS was preserved at a temperature of 4 °C in a refrigerator before the HTC treatment. Commercial grades HZSM-5 (Si : Al = 25) and USY molecular sieve (Si : Al = ∼10–15) were supplied by Damas Beta and Macklin, respectively.

### 2.2. HTC of sewage sludge

**Fig. 1:**
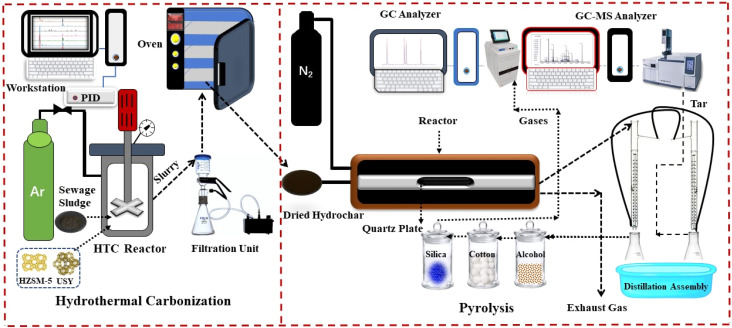
Schematic diagram of integrated HTC-Py setup.

HTC experiments were performed using an HT-250 J0 high-pressure batch reactor (HTLAB, Beijing, China) featuring an automatic electric heating system and a rotating stirrer, with a maximum operational pressure of 30 MPa and a temperature of 350 °C, as demonstrated in Fig. 1. In each set of experimental runs, 50 g of SS was mixed with varying concentrations (1%, 3%, 5%, and 7% wt.) of HZSM-5 and USY Zeolite catalysts and processed at 200 °C for 60 minutes in distinct series in accordance with our previous study.^39^ Following the closure of the reactor, the reaction chamber was purged with argon gas to sustain an O_2_-free environment, and the post-reaction products were collected through vacuum filtration to separate HC and process water. Upon completion of the HTC reaction, the reactor was cooled down, and the resultant HC was subjected to drying at 105 °C for 24 hours in an electric oven before analysis, with samples labeled as HC-Z1 to HC-Z7 for HZSM-5-derived products and HC-USY-Z1 to HC-USY-Z7 for USY derived products, where the numerical suffix denotes catalyst weight percentage. To guarantee uniformity and reproducibility, HTC experiments were carried out in a repeated manner.

### 2.3. Pyrolysis of catalyzed HC

A horizontal quartz tube reactor with a length of 1 m and an internal diameter of 50 mm was integrated into the reactor system. This reactor was fitted with an electrical heating element controlled by a temperature regulator and a K-type thermocouple. Following the placement of 5 grams of HC in a crucible at the reactor's midpoint, the sealed system was purged with nitrogen at a flow rate of 600 milliliters per minute. The thermal treatment was carried out at a heating rate of 10 °C per minute to achieve temperatures of 500 °C, 700 °C, and 900 °C. The reaction time at each temperature setpoint was 30 minutes. All experiments were carried out repeatedly to ensure the data could be reproduced accurately.

### 2.4. Analytical techniques

Multiple analytical techniques were employed to characterize HC samples prepared with different proportions of HZSM-5 and USY zeolite. The elemental composition, ash content, fixed carbon, and volatile matter were determined through ultimate and proximate analysis. The elemental composition was analyzed using a CHNS analyzer (Elemental vario EL, Germany). Fourier transform infrared spectroscopy (FTIR, Thermo Fisher Scientific, USA) was used to characterize the functional groups, while field emission scanning electron microscopy (FE-SEM, ZEISS, GeminiSEM 30) was adopted to analyze morphological features. Micromeritics ASAP2460 was used to perform low-pressure N_2_ adsorption at 77 K to evaluate surface properties. On the other hand, X-ray diffraction (XRD, Ultima IV, Japan) was utilized to investigate the material's crystalline structure. Thermal behavior was measured by thermogravimetric (TGA) analysis. Raman spectroscopy was performed at ambient temperature employing a LabRAM HR800-LS55 spectrometer (Horiba Jobin Yvon, France) featuring a silicon-based CCD detector, optical microscope, 532 nm Nd-YAG laser @ 5 mW power, maintaining a 20-second scanning duration, and 0.65 cm^−1^ spectral bandwidth. To study surface chemistry, XPS (ESCALAB 250Xi, USA) was applied with an Al Kα X-ray source (*hv* = 1486.6 eV) at 20 eV pass energy, 0.05 eV energy rise, and 0.1 s acquisition time, while its peak fitting was done using OriginPro 2024 software. Following the pyrolysis process, the composition of the resultant gases (H_2_, CH_4_, C_2_H_6_, C_2_H_4_, CO, CO_2_) was analyzed through a gas chromatograph (GC 990A) that was fitted with a thermal conductivity detector (TCD) and dual columns (5A and GDX-104) with Ar as the carrier gas. The analysis of resultant tar was carried out by employing a gas chromatography-mass spectrometry (GC-MS) technique (SHIMADZU QP2010 Ultra, with a 30-meter RESTEK capillary column, 0.25 mm internal diameter × 0.25 μm film thickness), maintaining the injection port temperature at 300 °C, and the initial GC heating protocol was set at 35 °C for 5 minutes.

## 3. Results and discussion

### 3.1. Physicochemical properties of catalyzed HC

#### 3.1.1. Ultimate and proximate analysis

**Fig. 2:**
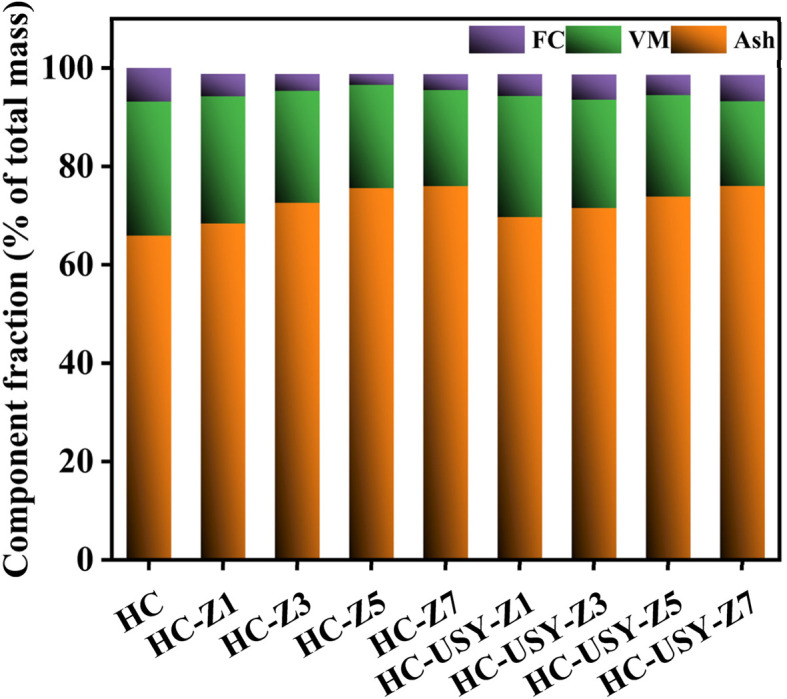
Component fraction of HC with increasing percentages of zeolite catalyst.

Findings from the elemental study indicated that higher concentrations of zeolite led to a decrease in carbon, nitrogen, and sulfur content, demonstrating the catalysts' effectiveness in removing heteroatoms, as presented in Table 1. The proximate analysis of HC comprises three primary components: fixed carbon (FC), volatile matter (VM), and ash, with varying proportions of zeolite catalysts illustrated in Fig. 2. The data indicate anticipated trends of reduced VM and high ash content with the increase in percentages of both zeolite catalysts following the trend as studied by Liu *et al.*^11^ Hydrogen and oxygen content reduction was ascribed to dehydration and decarboxylation reactions during HTC.^40^ The dissolution of inorganic nitrogen and the conversion of protein nitrogen to ammonium (NH_4_^+^–N) resulted in a decrease in nitrogen content.^11^ The increase in ash content within the HC can be explained by devolatilization, the decomposition of oxygenated functional groups in biopolymers, and the retention of minerals.^41^ This behavior corroborates findings from previous studies that examined HCs derived from municipal solid waste, SS, and miscanthus through proximate analysis.

**Table 1:** Ultimate analysis of HC with increasing percentages of zeolite catalysts^a^

Sample	Ultimate analysis (wt%, d)				
	C	H	N	S	O
HC	20.54 ± 0.110	2.46 ± 0.014	1.58 ± 0.008	0.35 ± 0.001	9.116 ± 0.048
HC-Z1	18.24 ± 0.108	2.12 ± 0.010	1.44 ± 0.007	0.35 ± 0.002	7.591 ± 0.038
HC-Z3	16.14 ± 0.092	2.03 ± 0.012	1.33 ± 0.007	0.29 ± 0.001	8.289 ± 0.047
HC-Z5	13.02 ± 0.072	1.74 ± 0.010	1.13 ± 0.005	0.24 ± 0.001	9.428 ± 0.051
HC-Z7	11.65 ± 0.060	1.43 ± 0.007	1.08 ± 0.005	0.21 ± 0.001	9.630 ± 0.049
HC-USY-Z1	17.58 ± 0.090	2.10 ± 0.010	1.44 ± 0.007	0.29 ± 0.001	7.410 ± 0.040
HC-USY-Z3	16.84 ± 0.085	1.91 ± 0.009	1.41 ± 0.007	0.28 ± 0.001	7.660 ± 0.038
HC-USY-Z5	14.98 ± 0.087	1.86 ± 0.009	1.35 ± 0.007	0.27 ± 0.001	8.400 ± 0.049
HC-USY-Z7	12.54 ± 0.070	1.64 ± 0.009	1.15 ± 0.006	0.25 ± 0.001	8.870 ± 0.046

a Oxygen (%) = 100 − (carbon (%) + hydrogen (%) + sulphur (%) + ash (%)), d = dry basis.

**Fig. 3:**
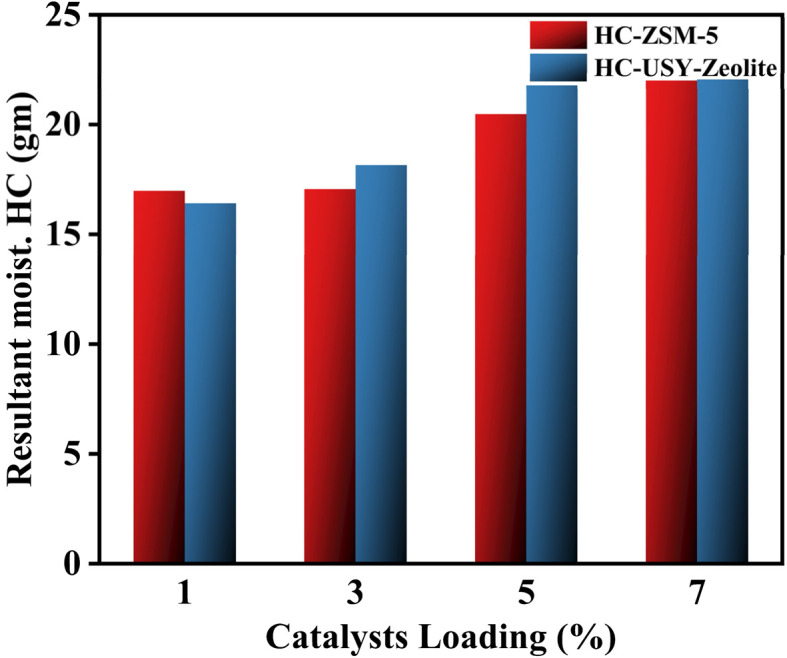
Mass of resultant moist. HC (gm) yield w. r. t. catalyst loading (%).

HC produced at varying temperatures contains a significant amount of ash, attributable to the elevated levels of inorganic matter and, consequently, the higher ratio of SS to zeolite during HTC. It consistently exhibited a significantly lower FC content compared to pure HC. In contrast to the simple HC, the FC content significantly differed from that in the zeolite-based HC. Furthermore, Fig. 3 illustrates the fraction of resultant HC as the percentages of zeolite catalysts increase. The yield shows a negligible decrease when zeolite catalysts are added in varying proportions, specifically at the 1% addition level. Nevertheless, as the percentages of zeolite catalysts rise, the yield shows a minimal increase from 20.481 g to 21.873 g. A higher catalyst percentage leads to interference from zeolite in the HTC process,^33^ potentially consuming some of the energy from the carbonization reaction and thereby affecting HC yield.

#### 3.1.2. Morphological and textural analysis

**Fig. 4:**
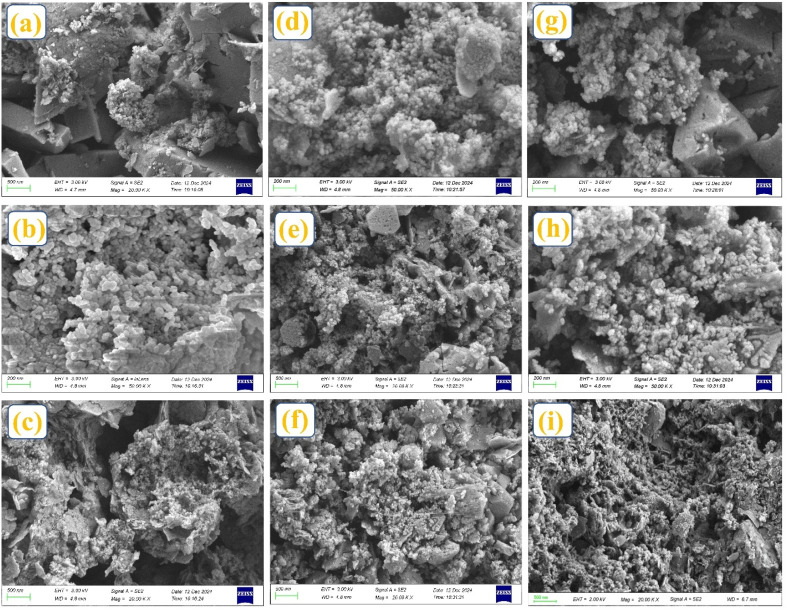
Morphology of (a) uncatalyzed HC and catalyzed HC w. r. t. increasing catalyst loading (b) HC-Z1, (c) HC-Z3, (d) HC-Z5, (e) HC-Z7, (f) HC-USY-Z1, (g) HC-USY-Z3, (h) HC-USY-Z5, (i) HC-USY-Z7.

SEM analysis was performed to investigate the systematic morphology, surface characteristics, and structural changes of HC at different zeolite catalyst concentrations, as depicted in Fig. 4(a–i). The uncatalyzed HC structure demonstrated enhanced fragmentation and porosity as a function of reaction temperature and residence time (Fig. 4(a)), attributed to the release of volatile gases during devolatilization and the breaking down of chemical bonds within the sludge.^42^ It is evident that at 1% HZSM-5 loading, HC-Z1 predominantly exhibited luminous polycrystalline aggregates characterized by uneven distribution and defined geometric configuration as depicted in Fig. 4(b). However, at a 3% HZSM-5 loading, catalyst particles exhibited a localized clustering in resultant HC while maintaining a relatively uniform distribution as demonstrated in Fig. 4(c). Meanwhile, there is a noticeable change in the HC-Z5, where zeolite aggregates cover more HC surface area and partly obscure the initial porous structure. However, the most dramatic evolution occurs at 7% loading of HZSM-5, where the resultant HC (HC-Z7) is marked by the formation of extensive nanosized particulate aggregates that create dense agglomerated structures, thereby significantly modifying surface topography.

Furthermore, the primary morphological structure of the HC samples was predominantly preserved across different catalyst concentrations, aligning with prior research.^43^ With notable variations, a comparable progression is seen for the USY-zeolite series. At 1% USY loading, zeolite particles in the HC product (HC-USY-Z1) exhibited a more cohesive integration within the HC matrix, as illustrated in Fig. 4(f). The 3% and 5% samples demonstrated a progressive formation of zeolite clusters while demonstrating superior overall accessibility compared to equivalent HZSM-5 samples. At 7% loading (HC-USY-Z7), notable agglomeration is observed; however, the boundaries between zeolite particles and the HC substrate are more clearly defined as depicted in Fig. 4(i).

**Fig. 5:**
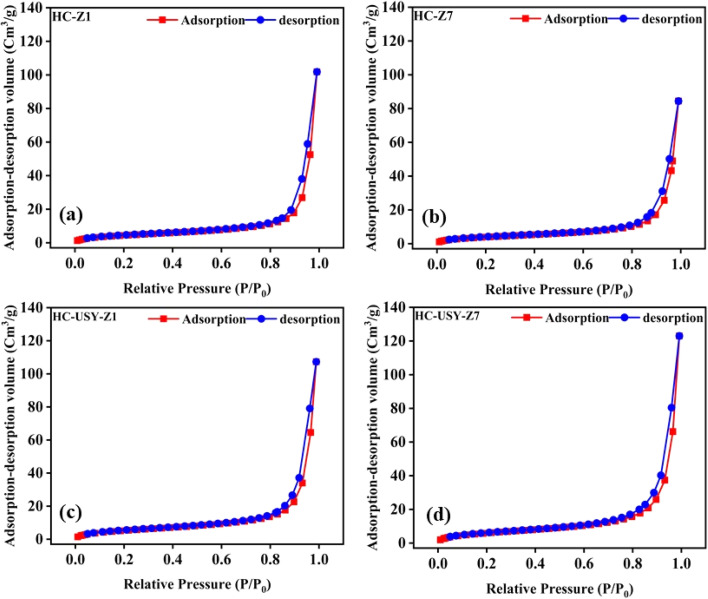
Adsorption/desorption isotherms of HC with increasing catalyst loading; (a) HC-Z1, (b) HC-Z7, (c) HC-USY-Z1, (d) HC-USY-Z7.

Brunauer–Emmett–Teller (BET) analysis was employed to assess the surface properties of HC with varying zeolite concentrations, as illustrated in Fig. 5 and Table 2. The analysis demonstrated N_2_ adsorption/desorption isotherms, specific surface area, and external surface area measurements with respect to the increasing ratio of catalyst. Table 2 indicates that both catalysts exhibited significant specific surface areas: 337 m^2^ g^−1^ for HZSM-5 and 831 m^2^ g^−1^ for USY zeolite.

**Table 2:** Surface area and pore analysis of HC with increasing percentages of catalysts

Sample	*S*_BET_ (m^2^ g^−1^)	*S*_external_ (m^2^ g^−1^)
HZSM-5	337.1224	20.9920
HC	19.1363	23.5047
HC-Z1	17.107	23.0266
HC-Z3	18.421	21.9517
HC-Z5	18.921	19.8554
HC-Z7	19.214	17.4201
USY-zeolite	831.0051	84.1625
HC-USY-Z1	20.4939	27.0554
HC-USY-Z3	22.8577	22.5958
HC-USY-Z5	23.5471	19.8754
HC-USY-Z7	25.5142	16.4514

Uncatalyzed HC exhibits a comparatively high *S*_external_ (23.5047 m^2^ g^−1^) and a modest *S*_BET_ of 19.1363 m^2^ g^−1^. In the HZSM-5 series, the BET surface area first decreases at 1% loading (HC-Z1: 17.107 m^2^ g^−1^) and then gradually increases as catalyst concentrations rise (HC-Z3 → Z5 → Z7: 18.421 → 18.921 → 19.214 m^2^ g^−1^), suggesting that the catalyst is contributing more to overall porosity even in the presence of partial pore blockage. Simultaneously, the external surface area decreases consistently with the rising HZSM-5 content (HC-Z1 → Z7: 23.0266 → 17.4201 m^2^ g^−1^), indicating ongoing agglomeration and diminished accessibility of external surfaces. The USY-zeolite series demonstrates consistently elevated BET surface areas compared to both uncatalyzed HC and the HZSM-5 series, showing a distinct positive correlation with catalyst loading (HC-USY-Z1 → Z3 → Z5 → Z7: 20.4939 → 22.8577 → 23.5471 → 25.5142 m^2^ g^−1^). This confirms a common mechanism of external surface modification across both catalyst types, as the external surface area decreases as the USY content increases (27.0554 → 16.4514 m^2^ g^−1^), similar to the HZSM-5 series. This difference cannot be explained solely by the Si/Al ratio, but also reflects the distinct pore architectures of the two materials. HZSM-5 possesses a medium-pore MFI structure (0.55 nm) with limited external accessibility and strong acidity, favoring selective dehydration and deoxygenation. Therefore, the observed catalytic trends are governed by the combined influence of pore structure, external surface area, and acidity, rather than by Si/Al ratio alone. It indicates that varying amounts of catalysts facilitated the production of agglomerated structures during HTC, obstructing the zeolite's outer surface and micropore channels.^44^ The potential cause of the reduction in overall surface area is that during HTC, organic materials are transformed into char, and the catalysis process results in the accumulation of carbonaceous deposits on its surface.^45^ This deposition can obstruct micropores and diminish the overall porosity of resultant HC. Previous research^46^ indicates that unaltered HZSM-5 and USY zeolite can undergo significant coke deposition, followed by a notable decrease in its micropore surface area with the more substantial percentage in certain instances. The N_2_ adsorption–desorption isotherms for both catalyst series exhibit characteristic Type IV patterns with H3 hysteresis loops according to IUPAC classification, indicating mesoporous structures with slit-like pores. At low relative pressures (*P*/*P*_0_ < 0.4), all samples show almost no N_2_ uptake, which suggests limited microporosity. At high relative pressures (*P*/*P*_0_ > 0.8), the samples show nearly vertical slopes approaching *P*/*P*_0_ = 1.0, which indicates that there are significant contributions from macropores. Regardless of differences in measured BET surface areas, analysis of the isotherms at different catalyst loadings shows that low-loading samples (HC-Z1, HC-USY-Z1) and their high-loading counterparts (HC-Z7, HC-USY-Z7) have similar adsorption capacities. Compared to the HZSM-5 series, the USY-catalyzed samples exhibit more pronounced hysteresis loops, particularly at higher loadings (HC-USY-Z7), indicating more sophisticated mesoporous networks. It appears that structural changes during HTC mainly affect pore accessibility rather than total adsorption capacity, as carbonaceous deposition favors external active sites before penetrating the microporous network. This effect is more noticeable in the highly microporous HZSM-5 than in the hierarchically porous USY-zeolite, as supported by the systematic alterations in BET and external surface areas and the negligible variations in the overall isotherm shape between low and high catalyst loadings. Typically, HC contains a considerable quantity of inorganic materials and ash, which rises during the HTC process. The existence of these inorganic elements can lead to a more compact structure within the HC, consequently decreasing its total porosity and surface area.^47^ Additionally, it encompasses the emission of volatile compounds from the feedstock. A considerable decrease in these volatiles results in a diminished overall volume and surface area of the resultant HC. Hence, the elimination of volatile organic matter could additionally lead to a denser structure, which in turn reduces porosity.

It is essential to highlight that to evaluate the impacts of HC with different zeolite catalyst ratios, a comprehensive set of characterization techniques, such as ultimate and proximate analysis, SEM, and BET, were conducted on all zeolite-based HC. The findings from these analyses revealed slight differences in the structural and chemical properties among the different proportions. Considering the restricted variation noted, we decided to concentrate our further detailed characterization efforts solely on the extreme proportions (*i.e.*, minimum and maximum zeolite catalyst concentrations) to minimize redundancy and improve the efficiency of our study. The supplementary analyses encompassed crystallinity, and thermal properties, yielding more detailed insights into the structural and thermal characteristics of the catalysts, thereby facilitating a more profound comprehension of catalyst behavior at these critical proportions.

#### 3.1.3. Functional group and surface chemistry analysis

**Fig. 6:**
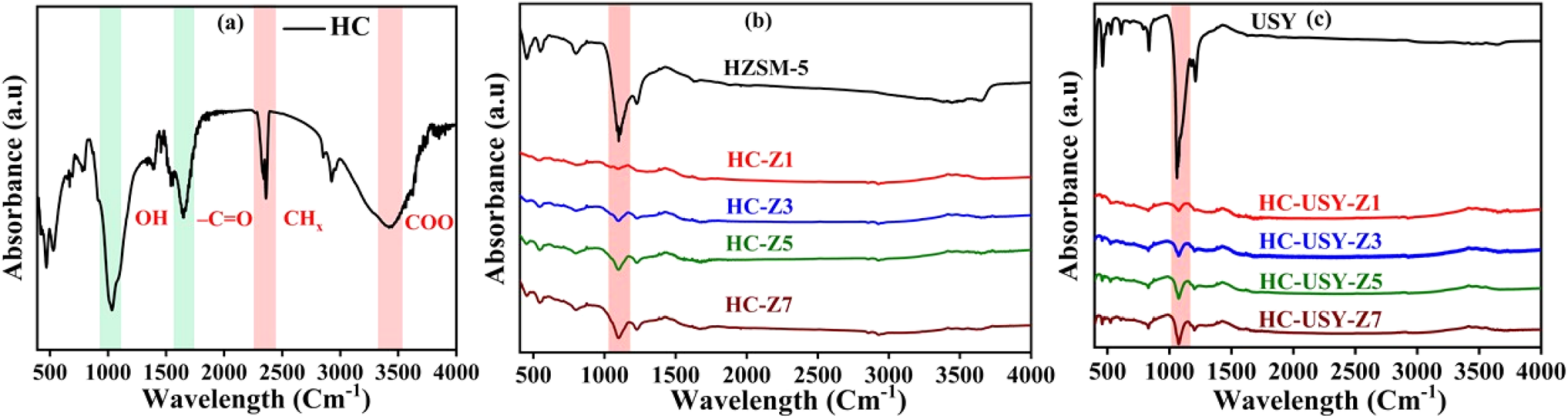
FTIR spectrum of (a) uncatalyzed HC, catalyst, and catalyzed HC with different catalysts loading (b) HZSM-5, HC-Z1 to HCZ7, (c) USY-zeolite, HC-USY-Z1 to HC-USY-Z7.

Fourier Transform Infrared Spectroscopy (FTIR) detects distinct functional groups in organic and inorganic substances by measuring their infrared radiation absorption within the range of 4000 cm^−1^ to 400 cm^−1^. Following Fig. 6(a) illustrates the FTIR bands of SS derived HC analogues to our previous study^48^ along with Fig. 6(b and c) demonstrating the catalyzed HC with varying percentages of zeolite catalysts. Utilizing zeolite catalysts individually and in various proportions yields significant insights into its chemical composition and structure.

A strong absorption band observed near 1645 cm^−1^ corresponds to C=O stretching vibrations of carbonyl groups such as ketones and amides, while a shoulder around 1540 cm^−1^ may be attributed to N–H bending (amide II) or aromatic C=C stretching^49^ as depicted in Fig. 6(a). The reduction in C=O intensity relative to O–H and COO bands indicates progressive decarboxylation and aromatic condensation during HTC,^50^ consistent with the elemental analysis trends discussed in Section 3.1.1 HZSM-5 and USY-zeolite demonstrated MFI structure peaks at 450 cm^−1^, 550 cm^−1^, 800 cm^−1^, 1100 cm^−1^, and 1220 cm^−1^ (internal tetrahedral T–O bending vibration, double rings, internal asymmetric stretch, and external asymmetric stretch) as illustrated in Fig. 6(b and c).^51,52^ In the case of the HZSM-5 catalyst, it displayed O–H groups at approximately 3605 cm^−1^ band indicating its presence in the framework of adsorbed water or aluminium, suggesting catalytic activity, as illustrated in Fig. 6(b). In the HZSM-5 sample, the detected peak at the 1630 cm^−1^ band indicates the existence of adsorbed H_2_O in the HZSM-5 catalyst sample. The asymmetrical stretching vibration at 796 cm^−1^ could be attributed Al_2_O_3_ or SiO_4_ in HZSM-5 lattices along with detection of stretching vibration near 554 cm^−1^ for the HZSM-5 catalyst. Comparable findings can be validated by ref. 53. Furthermore, no significant shifts in band positions between these two types of catalysts indicated no isomorphous substitution occurring in the zeolite framework. This behavior also reinforces the XRD of catalysts outlined in Section 3.1.4. Moreover, vibrations ranging between 3600–3200 cm^−1^ are generally linked to the stretching vibrations of hydroxyl groups (–OH) found in alcohols, phenols, and carboxylic acids. This suggests the existence of residual OH from the original organic matter in the SS. The observed vibration is linked to water absorption, potentially from adsorbed moisture or water released during carbonization. The spectral region from 2100 to 2300 cm^−1^ in FTIR analysis is generally related to the stretching vibrations of triple bonds. In our case, strong peaks in this region are less frequent than in other functional groups, attributable to the presence of unsaturated hydrocarbons, such as alkynes.

**Fig. 7:**
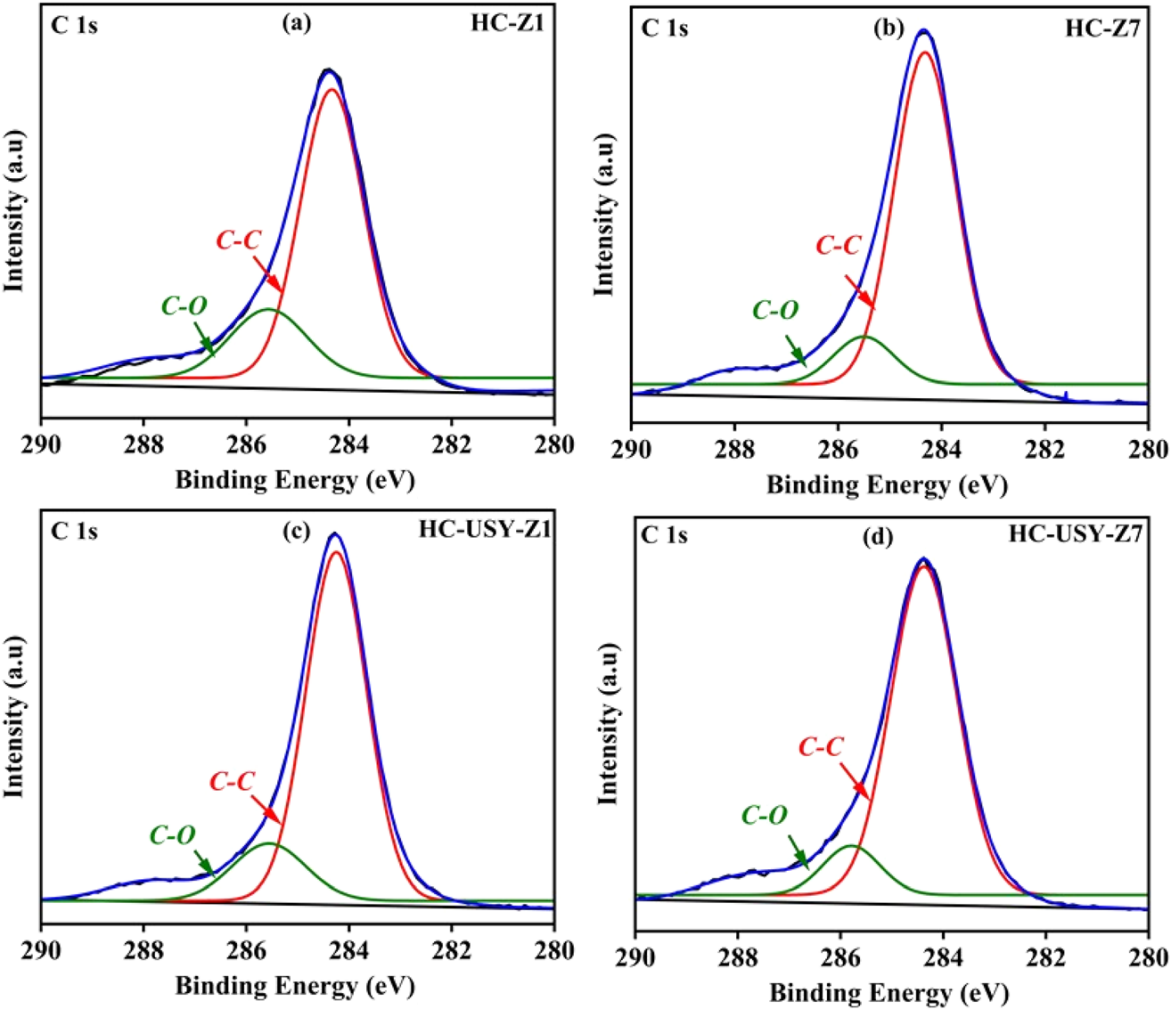
XPS spectra of catalyzed HC; (a) HC-Z1, (b) HC-Z7, (c) HC-USY-Z1, (d) HC-USY-Z7.

The XPS study resulted in essential information regarding the surface chemistry of catalyzed HCs. Table 3 presents a summary of the relative intensities of significant functional groups detected on the different HC surfaces. The identification of C–O (285.6 eV) and C–C (284.6 eV) bonds was established *via* the deconvolution of the C 1s spectra presented in Fig. 7(a–d). It revealed a progressive reduction in C–O functionalities accompanied by an increase in C–C bonding intensity, indicating surface reconstruction and enhanced carbon densification during HTC. This transformation was more pronounced in the USY-supported samples, which is consistent with its larger pore diameter and higher external surface areas depicted in Table 2. These physicochemical characteristics facilitate the diffusion of oxygenated intermediates and promote deoxygenation and aromatization reactions, leading to greater carbonization compared to the ZSM-5-based systems. The HC-Z1 sample demonstrated a bond distribution of 22.37% C–O, and 77.62% C–C, reflecting a significant carbon backbone content alongside moderate levels of oxygenation.

**Table 3:** Relative intensities of catalyzed HC with different proportions of zeolite catalysts

Samples	C–O	C–C
HC-Z1	22.37	77.62
HC-Z7	12.22	87.77
HC-USY-Z1	16.39	83.60
HC-USY-Z7	11.36	88.63

In contrast, HC-Z7 exhibited notable changes in its composition, with 12.22% C–O and 87.77% C–C bonds. This indicates substantial surface reconstruction at higher catalyst loadings, facilitating C–C bond formation while reducing C–O functionalities. The HC-USY series analysis suggests that HC-USY-Z1 exhibited 16.39% C–O, and 83.60% C–C bonds, reflecting a more excellent carbonyl content than HC-Z series. The HC-USY-Z7 sample exhibited an identical functional group distribution, characterized by a decrease in oxygenated species (11.36% C–O) and a substantial increase in C–C content (88.63%). This suggests that the USY zeolite catalyst facilitated aromatization and carbonization reactions, effectively maintaining the carbon backbone while selectively eliminating oxygenated groups. The observed trends in surface chemistry are consistent with the catalytic deoxygenation mechanisms, corroborating the results from the ultimate analysis, which indicated a reduction in oxygen content with the addition of the catalyst. The USY zeolite exhibited its strong behavior for maintaining carbon structures while effectively eliminating oxygen *via* decarboxylation and decarbonylation mechanisms, aligning with its established catalytic characteristics in HTC processes.

#### 3.1.4. Elucidating crystallinity and structural analysis

**Fig. 8:**
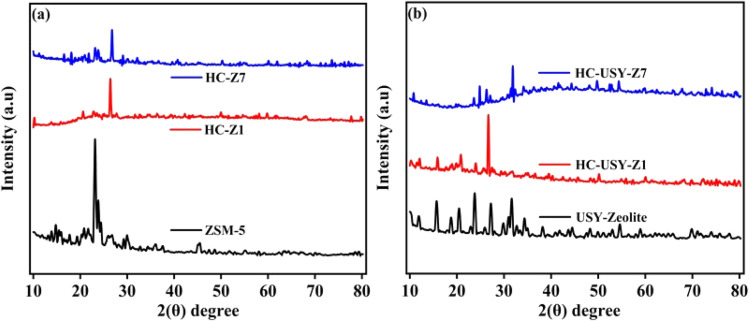
XRD of catalysts and catalyzed HC; (a) HZSM-5, HC-Z1, HC-Z7 (b) USY-zeolite, HC-USY-Z1, HC-USY-Z7.

Materials crystallinity is investigated through a diffraction pattern by employing X-ray diffraction (XRD) followed by significant insights into catalyzed HC, thereby highlighting their unique structural interactions when adding both catalysts. The spectra of HC with varying proportions of zeolite catalysts were analyzed to elucidate the influence of inorganic matter, as illustrated in Fig. 8(a and b). Diffraction patterns show that HTC does not affect HZSM-5's distinctive MFI framework peak at 2*θ* ≈ 23–24°, and that the crystalline structure of the zeolite remains unaffected in HC samples at both low (HC-Z1) and high (HC-Z7) concentrations as depicted in Fig. 8(a). This structural resilience verifies that HZSM-5 maintains its initial crystalline structure even in the extreme hydrothermal environment. However, the observed decrease in peak intensity with higher catalyst loading suggests partial pore blockage and surface coverage during HTC processing, which affects the accessibility of catalytic sites while maintaining structural stability. The lack of diffraction peaks associated with metal oxides in both catalysts suggests that the species are uniformly distributed at nanoscale dimensions, which is ideal for catalytic uses.^54,55^ The USY-zeolite series exhibits a more intricate incorporation behavior. The parent USY-zeolite displays a complex diffraction spectrum typical of its faujasite framework; however, these specific peaks show considerable distortion in the prepared HC samples (HC-USY-Z1 and HC-USY-Z7). The lack of distinctive diffraction peaks in HC-USY-Z samples indicates that Si/Al species are evenly distributed within the HC matrix at the nanometer scale,^56^ thereby enhancing catalytic accessibility.

These diffraction patterns indicate that HZSM-5 remains structurally independent during HC formation, while USY-zeolite integrates more into the carbonaceous matrix, which could contribute to their catalytic performance in TCC processes.

**Fig. 9:**
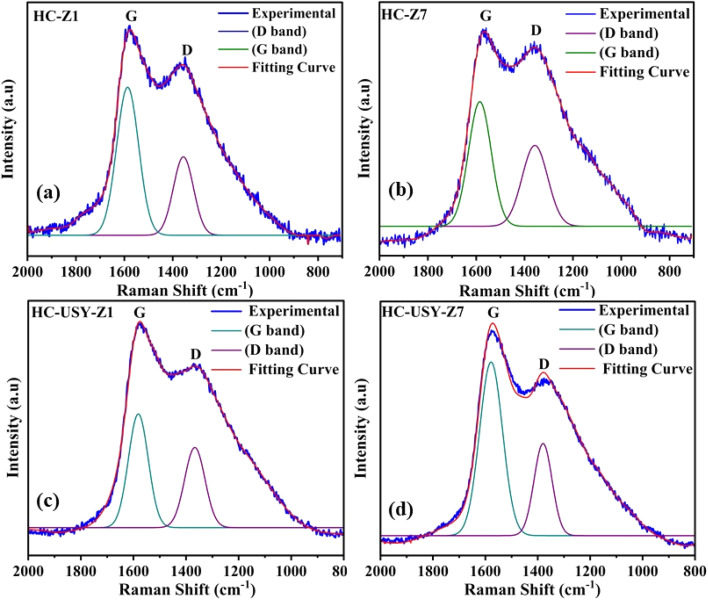
Raman spectra of catalyzed HC (a) HC-Z1, (b) HC-Z7, (c) HC-USY-Z1, (d) HC-USY-Z7.

Raman spectroscopy revealed essential carbon structural details of HC samples prepared under variable catalysts loading as demonstrated in Fig. 9(a–d). The spectra showed two main bands: the D band (≈1350–1380 cm^−1^), indicating disordered C structures, and the G band (≈1580–1600 cm^−1^), indicating sp^2^ graphitic carbon networks. Statistical ID/IG intensity ratios showed a consistent structural transformation across both catalysts. HC-Z1 had an ID/IG ratio of 0.48, suggesting a relatively ordered C with a graphitic carbon structure. This ratio correlates with the proximate and ultimate analyses, where HC-Z1 (Fig. 9(a)) preserved 18.24% C and had a medium surface redevelopment, as also shown by XPS (77.62% C–C bonds).

In contrast, HC-Z7 (Fig. 9(b)) had a 52% higher ID/IG ratio of 0.73 than its low-loading predecessor, indicating structural disorder. This modification towards a more disorganized C structure is directly linked to the massive drop in activation energy (from 27 kJ mol^−1^ to 5.5 kJ mol^−1^) as identified by TGA analysis. This enhanced structural disorder created more reactive sites, improving catalytic performance during pyrolysis, as demonstrated in Section 3.2.1. An interesting behavior was observed in the USY zeolite series, followed by a structural disorder of HC-USY-Z1's (Fig. 9(c)) with an ID/IG ratio of 0.72 at 1% catalyst loading. This finding clarifies the phenomena of temperature-dependent H_2_ production and the peculiar gaseous product distribution during pyrolysis. This disorganized C structure made it easy for H_2_ to release, even at low temperatures. Of all the samples, HC-USY-Z7 (Fig. 9(d)) exhibited the lowest ID/IG ratio at 0.42, which was the most remarkable. At high USY loading, this structural arrangement is different from the HZSM-5 catalyst in a mechanistic sense. A high H_2_ yield and a CO production at 900 °C are both attributed to HC-USY-Z7's highly ordered C structure as explained in Section 3.2.1. The widening of the D band, which indicates the introduction of defects and amorphization of the carbon matrix, was observed in HZSM-5 samples as the catalyst loading increased. Catalyst interaction caused minor shifts in the G band, from 1585 cm^−1^ in HC-Z1 to 1592 cm^−1^ in HC-Z7. In HC-USY-Z7, the distinctive shoulder around 1620 cm^−1^ verified the development of specialized graphitic structures with well-placed defects, enhancing catalytic performance without sacrificing structural integrity. The widening of the D band in samples with high catalyst ratio signifies an elevation in structural defects and amorphous carbon content. A minor displacement in the G band position from 1585 cm^−1^ in HC-Z1 to 1592 cm^−1^ in HC-Z7 indicates stress within the carbon lattice caused by the catalyst. The existence of a minor shoulder near 1620 cm^−1^ (D band) in the HC-USY-Z7 sample further substantiates the formation of defect-induced graphitic structures. The Raman results augment the XRD findings by demonstrating that the catalysts facilitate structural reconfiguration within the carbon matrix, leading to a more disordered carbon structure with heightened defect density at elevated catalyst loadings. The observed spectral characteristics correspond with prior studies on hydrothermally carbonized biomass materials, validating that zeolite catalysts affect both the surface chemistry and the intrinsic carbon structure of the resultant HC. The spectroscopic patterns indicate that the type and loading of the catalyst have distinct effects on the development of carbon nanostructures during HTC process. HZSM-5 exhibits increased structural disorder at elevated loadings, which may improve the availability of reactive sites. In contrast, USY-zeolite promotes a more controlled structural transformation through selective defect openings. The observed variations in thermal reactivity and product selectivity during subsequent pyrolysis are probably caused by these clear carbon structural changes.

#### 3.1.5. Unveiling the thermal characteristics of catalyzed HC

**Fig. 10:**
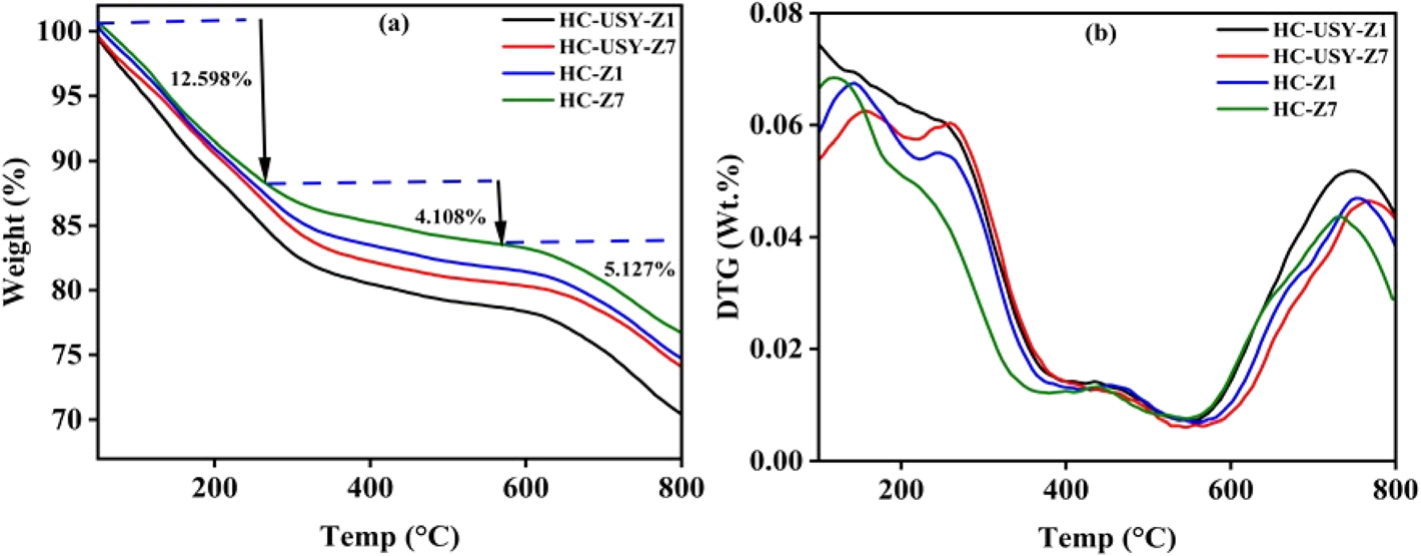
Thermal analysis; (a) TGA of HC-Z1, HC-Z7, HC-USY-Z1, HC-USY-Z7 and (b) DTG of HC-Z1, HC-Z7, HC-USY-Z1, HC-USY-Z7.

Thermodynamic studies of catalyzed HC provided insights into its structural makeup and potential reactivity. Thermogravimetric analysis (TGA) and its derivative (DTG) effectively identified the decomposition characteristics of HC at various catalyst loadings, highlighting specific stages of mass change as demonstrated in Fig. 10(a and b). TGA profile demonstrated specific phases: volatilization of substances (56–250 °C), FC combustion, and transformation of char into gas (265–800 °C), illustrated in Fig. 10(a). Upon increasing temperature from 56 °C to 268 °C, the HC exhibited a substantial mass loss of approximately 11 to 12%. Generally, HTC decomposes volatile substances and increases the quantity of ash in the HC, resulting in mass loss. A more pronounced effect was due to an increased proportion of zeolite affecting total breakdown, as illustrated in Fig. 10(b). Previous research^57^ demonstrated that the HTC of SS preserved its stability, necessitating increased energy for breakdown.

DTG identified significant transition points at which decomposition rates attained their peak intensity by categorizing the dynamics of weight loss into distinct stages, each indicating particular structural changes. The initial phase, occurring between 30 °C and 100 °C, is predominantly attributed to moisture evaporation. The following principal mass loss phase, ranging from 200 °C to 600 °C, is linked to the degradation of the primary constituents of SS. In this phase, protein degradation occurs throughout a wide temperature range of 250 °C to 800 °C, indicating the intricate processes involved in HTC. The peak rate of weight loss is noted near the inflection point, occurring between 56.12 °C and 271 °C, associated with a weight reduction of roughly 12 to 14% as demonstrated in Table 4.

**Table 4:** Thermal behavior of catalyzed HC with increasing proportions of zeolite catalysts

Thermal stages	HC-USY-Z1	HC-USY-Z7	HC-Z1	HC-Z7
First decomposition (*T*_i_, °C)	56.12	57.18	56.13	51.455
1st weight loss (%)	14.032	11.865	12.205	12.59
Second decomposition (*T*_max_, °C)	265.56	268.51	265.566	271.511
2nd weight loss (%)	12.598	6.558	5.231	4.747
Final decomposition (*T*_Final_, °C)	796.12	791.19	796	796.206
Final weight loss (%)	6.572	5.686	7.601	6.704

The location of these inflection points either remains relatively stable or shifts marginally in the presence of a catalyst, indicating that the catalyst may affect the process or mechanism of degradation without substantially changing the temperature range for the most significant weight loss. The inflection point shifted from 265 to 271 °C with the addition of 1% and 7% in both catalysts. The ultimate weight reduction phase noted from (265–796 °C) pertains to protein carbonization, which constitutes the principal component of SS. Overall, the general curves remained consistent across varying catalyst proportions; nevertheless, significant mass losses were evident. These results can be confirmed by ref. 58.

This study also examines essential kinetic parameters, specifically *E*_a_ (activation energy) and *A* (exponential factor), as defined by the Arrhenius equation:
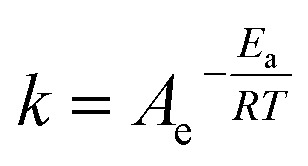
where, *k* = rate constant, *A* = pre-exponential factor, *E*_a_ = activation energy, *R* = universal gas constant, *T* = absolute temperature.

The parameters offer insights into thermal decomposition processes and energy conversion efficiency. In the context of HTC, it denotes the energy barrier that must be surpassed for the thermal decomposition of organic materials in HC. It indicates that an increase in catalysts loading of HZSM-5 significantly decreased activation energy of their resultant HC (Table 5), suggesting that less energy is necessary for the reaction to occur. This enhancement in reactivity contributes to the improved efficiency of HC as a fuel. However, in the case of USY zeolite, the activation energy does not appear to decrease as presented in Table 5. The consistent activation energy is attributed to its stable framework composition. The USY zeolite demonstrates exceptional framework stability owing to its low aluminum concentration and elevated silica-to-alumina ratio. This stability reduces structural alterations during the reaction, resulting in uniform kinetic characteristics, such as activation energy. Secondly, the uniformity of active sites inside USY zeolite persists throughout the reaction process.^59^ This uniformity guarantees that the energy threshold for the reaction remains stable, as every active site contributes equally to the catalytic activity. The exponential factor (*A*) decreases, indicating a reduction in the frequency of collisions between reactant molecules and their orientation during these interactions. The differences in the exponential factor indicate variations in the complexity of the reactions. A more prominent exponential factor suggests increased reaction pathways or a greater likelihood of effective collisions between reactants.^60,61^ Typically, HC demonstrates lower exponential factors than raw SS, attributable to their modified structural properties following HTC. This reduction suggests that although HCs may decompose more quickly due to lower activation energy (*E*_a_), they may also exhibit simpler reaction mechanisms. HCs generally demonstrate lower activation energies due to improved carbonization and decreased volatile matter content, as discussed in Section 3.1.1.

**Table 5:** Evaluation of kinetics and their respective parameters

Samples	*E*_a_ (kJ mol^−1^)	*A*
HC-Z1	27	0.004
HC-Z3	15.26	0.107
HC-Z5	10.52	0.179
HC-Z7	5.5	0.213
HC-USY-Z1	26.9	0.001
HC-USY-Z3	26.9	0.0017
HC-USY-Z5	26.9	0.0020
HC-USY-Z7	26.9	0.0023

This change suggests that the HCs exhibit increased reactivity during carbonization, thereby promoting easier decomposition at intermediate temperatures. The values for activation energy and exponential factor exhibit significant variability influenced by feedstock composition and HTC conditions. Previous studies indicate that HCs from various feedstocks display different kinetic behaviors attributable to differences in their chemical structures and thermal stability.^62^

### 3.2. Strategic thermal decomposition: pyrolytic behavior of catalyzed HC

Pyrolysis can be performed at moderate (≤300 °C) and elevated (>300 °C) temperatures depending upon the desired yield. Dehydration reaction is promoted at low temperatures, yielding biochar. In contrast, high temperatures (>300 °C) facilitate secondary reactions such as thermal cracking, transforming biomass into bio-oils and gases (CO, CO_2_, CH_4_, H_2_), enabling process optimization according to the desired final products.

#### 3.2.1. Temperature-dependent gaseous products selectivity from catalyzed HC

**Fig. 11:**
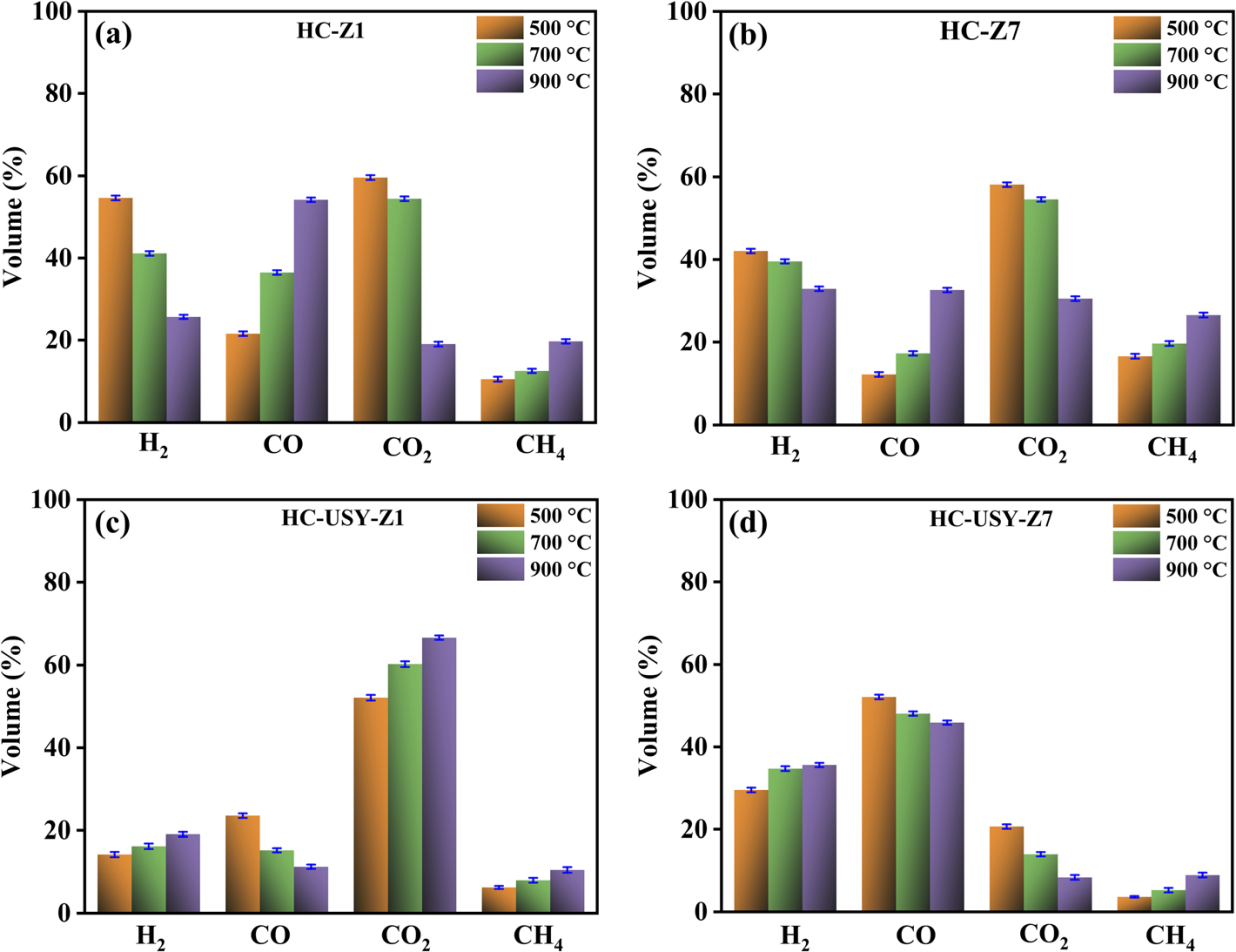
Effects of various temperatures on the gaseous composition of catalyzed HC; (a) HC-Z1, (b) HC-Z7, (c) HC-USY-Z1, (d) HC-USY-Z7.

Fig. 11 illustrates the gaseous products (H_2_, CO, CO_2_, and CH_4_) distribution resulting from the catalyzed HC's pyrolysis at three temperature levels (500 °C, 700 °C, and 900 °C). In the case of HC-Z1, as demonstrated in Fig. 11(a), H_2_ production highlights a significant temperature dependence, with yields declining from 54.64% at 500 °C to 25.65% at 900 °C. In contrast, CO generation exhibits a positive trend with temperature, rising from 21.56% at 500 °C to 54.18% at 900 °C. This might be because of increased reaction temperature, which reduces the reactivity of the water gas shift reaction.^63^ However, with increasing temperature, CO_2_ yields fall from 59.6% at 500 °C to 19% at 900 °C, whereas CH_4_ production moderately increases from 10.57% to 19.68% for the same temperature range. For HC-Z7, as shown in Fig. 11(b), H_2_ production exhibits an identical declining trend, decreasing from 42.05% at 500 °C to 32.9% at 900 °C, although the decrease in yield is less pronounced than that observed with the 1% loading. The production of CO rises significantly from 12.25% to 32.65% with increasing temperature, whereas the yields of CO_2_ decline markedly from 58.13% at 500 °C to 30.52% at 900 °C. The formation of CH_4_ shows a steady increase from 16.54% to 26.54% as temperature rises propagating it a beneficial conversion feedstock towards methanol.^64^ The distinct structural features and response mechanisms of the HZSM-5 catalyst series account for the divergent behavior, thereby suggesting a mechanistic transition from dehydrogenation to decarbonylation processes, as higher temperatures favor CO formation over H_2_ production at lower catalyst loading.

This is related to the proven observation that the catalyst has a strong Brønsted acidity and shape selectivity, which make it easier to break C–O bonds at higher temperatures.^65^ Reactive sites that are particularly favorable to pathways for CO formation are created by the increased structural disorder, as also observed in Raman analysis (ID/IG ratio: 0.48 → 0.73). It was also found that with the increase in the loading of HZSM-5, the release of H_2_ gas was decreased while increasing the production of CH_4_. This aligns with earlier research that found HZSM-5's unique channel structure (5.4–5.6 Å) affects product selectivity during biomass thermal conversion.^65^ Meanwhile, Increased catalyst loading seems to mitigate temperature impacts on H_2_ production while promoting CH_4_ generation at higher temperatures, probably due to enhanced catalytic hydrogenation of carbon species.

On the other hand, as the temperature increased, the HC-USY-Z1 sample established a distinctive behavior, with H_2_ yields increasing from 14.21% to 19.05%, whereas CO displays an inverse trend, decreasing from 23.54% to 11.25%, as demonstrated in Fig. 11(c). CO_2_ production rises from 52.12% to 66.67% with increasing temperature, while CH_4_ experiences a slight increase from 6.25% to 10.48%. The USY catalyst at 1% loading facilitates various reaction mechanisms, promoting CO_2_ formation at elevated temperatures instead of CO, potentially by enhancing water–gas shift reactions. In the case of HC-USY-Z7, as depicted in Fig. 11(d), H_2_ production exhibits a moderate increase with temperature, rising from 29.52% to 35.6%, whereas CO yields experience a slight decline from 52.12% to 45.9%. CO_2_ production declines markedly with increasing temperature, from 20.63% to 8.38%, while CH_4_ formation rises from 3.65% to 8.95%. Increased USY loading facilitates H_2_ production at higher temperatures while preserving significant CO yields, suggesting synergistic effects that improve overall syngas (H_2_ + CO) production. The reason behind this unique pattern is that, in comparison to HZSM-5, USY has an open framework and a greater pore diameter (7.4 Å), which allow multiple reaction paths.^66^ Notably, at higher loading of USY, H_2_ production is increased while maintaining high CO yields at all temperatures, indicating synergistic effects optimizing H_2_+CO production. This behavior is in line with the finding from Raman analysis about ordered carbon structure seen at higher USY loadings (ID/IG ratio dropping to 0.42). This creates a structure that stabilizes carbonaceous species while promoting H_2_ release more easily. The findings indicate that zeolite type and loading percentage significantly affect the temperature-dependent evolution of gaseous products during pyrolysis. The HZSM-5 catalyst selectively enhances CO production at elevated temperatures, particularly under lower loadings, whereas USY zeolite favors H_2_ generation with rising temperatures, especially at higher loadings. The reduction in CO_2_ yields across most samples, except for HC-USY-Z1, indicates that higher temperatures promote the Boudouard reaction, transforming CO_2_ into CO.^67^ The increased CH_4_ production observed in all samples at elevated temperatures suggests a rise in methanation reactions attributed to the heightened catalytic activity of both zeolite types.^68^

#### 3.2.2. Temperature-dependent tar products selectivity from catalyzed HC

**Fig. 12:**
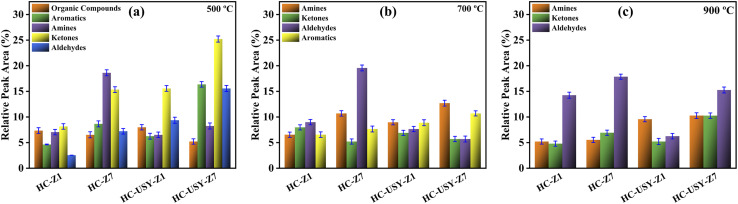
Effects of various temperatures on tar composition of all catalyzed HC; HC-Z1, HC-Z7, HC-USY-Z1, HC-USY-Z7 (a) 500 °C, (b) 700 °C, (c) 900 °C.

Pyrolytic tar products from catalyzed HC samples at 500 °C, 700 °C, and 900 °C are depicted in Fig. 12(a–c) HC samples. At 500 °C, HC-Z1 has a balanced composition of organic compounds (7.34%), aromatics (4.63%), amines (7.04%), ketones (8.15%), and aldehydes (2.54%), while HC-Z7 favors nitrogen compounds (18.62% amine) and 15.33% ketones as illustrated in Fig. 12(a). However, HC-USY-Z7 gave significantly higher yields of ketones (25.21%), aldehydes (15.56%), and aromatic content (16.36%), possibly because of USY's large pore diameter promoting aromatic formation, as in line with the findings by Boxiong *et al.* (2007).^66^ This suggests that USY loading boosts carbonyl compound formation at 500 °C. At 700 °C, the pyrolytic product distribution was evenly distributed in HC-Z1, while the same for HC-Z7 transformed significantly, yielding 10.69% amine, 19.56% aldehyde, and 5.21% ketone formation. HC-USY-Z1 has moderate levels in all categories, while HC-USY-Z7 has 12.69% amines and 10.69% ketones with reduced aromatics, demonstrating secondary cracking of organic vapors consistent with the results as reported by Vamvuka *et al.* (2011).^69^

The increased gas yields in Fig. 11(d) match the reduction in aromatics at 700 °C for HC-USY-Z7 compared to 500 °C, indicating gaseous product transformation. Aldehydes dominate all samples at 900 °C, as evident from Fig. 12(c), suggesting that high-temperature pyrolysis cracks larger molecules to increase aldehyde production. Aldehydes are the highest at 17.86% in HC-Z7, while amines (10.26%), ketones (10.25%), and aldehydes (15.26%) are more balanced in HC-USY-Z7. Both USY samples' elevated amine content at 900 °C matches their nitrogen content from ultimate analysis as documented in Section 3.1.1 (Table 1), indicating that USY catalysts remove nitrogen less efficiently than HZSM-5 at high temperatures. The results show that zeolite type and loading percentage affect temperature-dependent tar composition changes. Higher loadings of HZSM-5 increase aldehyde formation at high temperatures, while USY zeolite distributes nitrogen and oxygen-containing compounds more evenly. The systematic decrease in oxygen-containing compounds with higher HZSM-5 loading matches its enhanced deoxygenation capacity in the ultimate analysis, producing cleaner tar with reduced oxygen functionalities, which is beneficial for downstream applications that require low-oxygen bio-oils.

## 4. Conclusion

This research presented key insights into the zeolites-mediated HTC of SS, contributing towards the reverse engineering of hazardous waste into renewable energy generation. By strategically adding selective catalysts directly during the HTC phase, the derived HC's physiochemical structure was rearranged in a unique way by demonstrating specific reactivity profiles and the ability to transform into desired products. The study of HZSM-5 and USY zeolite catalysts at different concentrations (1–7%) established notable effects on HC characteristics and subsequent pyrolysis efficiency. Physicochemical characterization indicated that increased catalysts loading significantly reduced carbon, nitrogen, and sulfur contents *via* dehydration, decarboxylation, and denitrification processes. SEM analysis demonstrated progressive morphological changes from discrete crystalline structures at low catalyst loading to extensive agglomerated formations at higher concentrations. XPS and FTIR analyses confirmed significant surface reconstruction, revealing distinct patterns among different catalyst types. The thermal analysis demonstrated that HZSM-5 markedly decreased the activation energy of derived HC from 27 kJ mol^−1^ to 5.5 kJ mol^−1^ at higher concentrations, inducing improved structural disorder (ID/IG ratio: 0.48 → 0.73) by restructuring its carbon matrix. In contrast, USY zeolite exhibited a stable activation energy of 26.9 kJ mol^−1^ across all concentrations, attributable to its controlled defect engineering on derived HC. When subjected to temperature-dependent pyrolysis (500–900 °C), the pre-conditioning has a cascading effect, causing HZSM-5-modified HCs to produce H_2_ (54.64%) at lower temperatures and CO-dominated streams (54.18%) at higher ones. At the same time, USY-based HCs show great promise for producing syngas; specifically, HC-USY-Z7 produces optimized H_2_/CO ratios ideal for Fischer–Tropsch synthesis. This catalytic approach is further reinforced by tar analysis, which indicated that HC-USY-Z7 at 500 °C generated valuable aromatics (16%) and ketones (25%), whereas HC-Z7 at 900 °C resulted in the highest aldehyde content (17%) with decreased nitrogen compound. These results provide a solid foundation for future research into catalytic HTC mechanisms and open up new avenues for “selective” HC conversion pathways by carefully choosing catalysts, thereby advancing sustainable waste management practices and renewable energy technology.

## Author contributions

MR: conceptualization, methodology, investigation, data curation, formal analysis, writing – original draft, visualization. XZ: conceptualization, supervision, project administration, writing – review & editing. MAN: co-supervision, funding acquisition, writing – review & editing, AL, MSA, HZ, MFA, MKJ, MW: writing – review & editing.

## Conflicts of interest

The authors declare that they have no known competing financial interests or personal relationships that could have appeared to influence the work reported in this paper.

## Data Availability

The datasets supporting the findings of this study are available from the corresponding author upon request. All data generated or analyzed during this study are included in the article.
